# Active Zone Protein Bassoon Co-Localizes with Presynaptic Calcium Channel, Modifies Channel Function, and Recovers from Aging Related Loss by Exercise

**DOI:** 10.1371/journal.pone.0038029

**Published:** 2012-06-06

**Authors:** Hiroshi Nishimune, Tomohiro Numata, Jie Chen, Yudai Aoki, Yonghong Wang, Miranda P. Starr, Yasuo Mori, John A. Stanford

**Affiliations:** 1 Department of Anatomy and Cell Biology, University of Kansas Medical School, Kansas City, Kansas, United States of America; 2 Department of Molecular & Integrative Physiology, University of Kansas Medical School, Kansas City, Kansas, United States of America; 3 Department of Synthetic Chemistry and Biological Chemistry, Graduate School of Engineering, Kyoto University, Katsura, Kyoto, Japan; Virginia Commonwealth University Medical Center, United States of America

## Abstract

The P/Q-type voltage-dependent calcium channels (VDCCs) are essential for synaptic transmission at adult mammalian neuromuscular junctions (NMJs); however, the subsynaptic location of VDCCs relative to active zones in rodent NMJs, and the functional modification of VDCCs by the interaction with active zone protein Bassoon remain unknown. Here, we show that P/Q-type VDCCs distribute in a punctate pattern within the NMJ presynaptic terminals and align in three dimensions with Bassoon. This distribution pattern of P/Q-type VDCCs and Bassoon in NMJs is consistent with our previous study demonstrating the binding of VDCCs and Bassoon. In addition, we now show that the interaction between P/Q-type VDCCs and Bassoon significantly suppressed the inactivation property of P/Q-type VDCCs, suggesting that the Ca^2+^ influx may be augmented by Bassoon for efficient synaptic transmission at NMJs. However, presynaptic Bassoon level was significantly attenuated in aged rat NMJs, which suggests an attenuation of VDCC function due to a lack of this interaction between VDCC and Bassoon. Importantly, the decreased Bassoon level in aged NMJs was ameliorated by isometric strength training of muscles for two months. The training increased Bassoon immunoreactivity in NMJs without affecting synapse size. These results demonstrated that the P/Q-type VDCCs preferentially accumulate at NMJ active zones and play essential role in synaptic transmission in conjunction with the active zone protein Bassoon. This molecular mechanism becomes impaired by aging, which suggests altered synaptic function in aged NMJs. However, Bassoon level in aged NMJs can be improved by muscle exercise.

## Introduction

Synaptic transmission at the adult NMJs initiates by the Ca^2+^ influx through the P/Q-type VDCCs [1], [2] and synaptic vesicle fusion at the active zones [3]. Based on studies of NMJs and other synapses, the essential VDCCs for synaptic transmission have been estimated to localize at or in the close vicinity of active zones [2], [4]–[8]. An anatomical confirmation of these analyses is best suited in large synapses like the mammalian NMJs, but the relative location of the P/Q-type VDCCs and the NMJ active zones has not been revealed. The published immunohistochemistry studies by others show relatively diffuse distribution of P/Q-type VDCCs covering the entire presynaptic terminals of rodent NMJs [9]–[12]. This diffuse distribution of P/Q-type VDCCs in the presynaptic terminals is somewhat unexpected considering the discrete and punctate distribution of active zones in rodent NMJs detected by electron microscopy [13] and immunohistochemistry [14]–[16]. Thus, we first asked whether the P/Q-type VDCCs localize at the NMJ active zones. In relation to the accumulation of VDCCs at active zones, we and others have shown that the VDCCs and active zone proteins form protein complexes [14], [17]–[24]. We have shown that VDCC ß subunit and Bassoon interact for organizing the NMJ active zones [14]. However, the effect of the interaction between the P/Q-type VDCC and Bassoon on the channel function is not known. Therefore, we tested P/Q-type VDCCs using patch-clamp recording and demonstrated that the interaction of P/Q-type VDCCs and Bassoon modifies the VDCC function. This modification has the potential to play an important role in synaptic transmission at NMJs. If this interaction is essential for NMJ synaptic transmission, our recently findings of attenuated Bassoon protein levels in aged mouse NMJs may have deleterious effects on the NMJ function [15]. This view is consistent with the physiological alterations recorded at aged NMJs by others [25]–[27] and may be related to denervation of aged NMJs [25], [28]–[30]. Thus, it prompted us to seek ways to ameliorate the loss of Bassoon in the aged NMJs. We attempted exercising aged rodents because beneficial effects of exercise intervention for the nervous system have been described previously [31]–[35]. We identified that Bassoon level can be recovered in aged NMJs by muscle training.

## Results

### P/Q-type VDCCs Localize at the NMJ Active Zones

The starting point for this study was our previous finding that presynaptic VDCCs are essential for organizing active zones, and function as scaffolding proteins that anchor active zone proteins at presynaptic terminals [14], [16]. In these studies, we have demonstrated that VDCCs utilize their cytosolic domain to bind active zone proteins, which localize as discrete small puncta in NMJs. However, the relative location of P/Q-type VDCCs and the active zone proteins have not been analyzed in the published immunohistochemistry studies by others [9]–[12]. Furthermore, these staining patterns of P/Q-type VDCCs in NMJs were different from the discrete punctate staining pattern of active zone proteins that we identified [14]–[16]. Thus, we started by examining the distribution pattern of P/Q-type VDCCs in NMJs. We focused on P/Q-type VDCCs because adult NMJs utilize mainly this VDCC for synaptic transmission [1].

**Figure 1:**
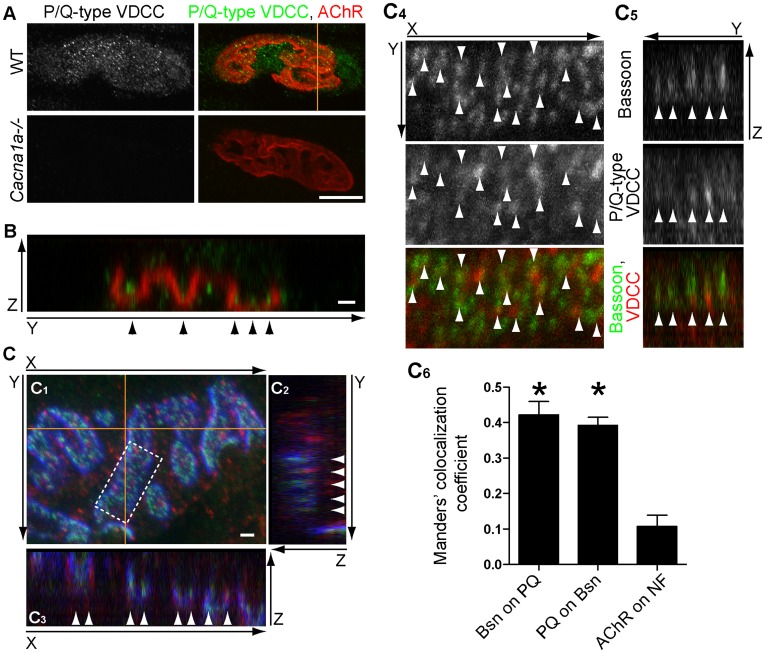
P/Q-type VDCCs localize at the NMJ active zones. (A) P/Q-type VDCCs stained with an antibody against the α subunit (Cav2.1, green) in NMJs of sternomastoid muscle from postnatal day 15 wild-type mice stained with Alexa Fluor 594-labeled α-bungarotoxin to label the acetylcholine receptors (AChR, red). The immunoreactivity is absent in the NMJ area of the littermate P/Q-type VDCC knockout mice (*Cacna1a^−/−^*) demonstrating the specificity of the immunohistochemical signals. The same result was confirmed in four independent litters. (B) P/Q-type VDCCs were detected inside the primary gutter of endplates labeled with α-bungarotoxin, where motor nerve terminals reside (arrowheads). An YZ-orthogonal view (a single optical plane) of a region indicated by an orange line in A is shown. The nerve is placed toward the top of the Z-axis and the muscle toward the bottom. Some P/Q-type VDCC signals were also detected in the muscle side. (C) P/Q-type VDCCs aligned with the active zone protein Bassoon in NMJs (arrowheads). Sternomastoid muscle of postnatal day 21 wild-type mice was stained using an anti-P/Q-type VDCC α subunit antibody (red), an anti-Bassoon antibody (green), and Alexa Fluor 647-labeled α-bungarotoxin (blue). The maximal projected XY-view of confocal Z-stack is shown in C_1_. Panels C_2_ and C_3_ show YZ- and XZ-orthogonal views (a single optical plane) at the positions indicated by the orange lines in C_1_. Panels C_4_ and C_5_ show a magnified region of C_1_ indicated by the dotted-box (C_4_) and orange line inside the dotted box (C_5_). Many Bassoon and P/Q-type VDCCs signals (white arrowheads) align in the XY-views (C_4_) and YZ-orthogonal views (C_5_, single optical plane). In C_5_, the nerve is placed towards the top, and muscle is placed towards the bottom. (C_6_) Colocalization analysis of Bassoon and P/Q-type VDCC within NMJ presynaptic terminals of postnatal day 21 wild-type mice by the Manders’ coefficients (M). Some degree of colocalization of these proteins was indicated by the significantly higher Manders’ coefficients M values for Bassoon overlapping with P/Q-type VDCC (0.42±0.04, 3 NMJs, Bsn on PQ) and P/Q-type VDCC overlapping with Bassoon (0.39±0.02, 3 NMJs, PQ on Bsn) compared to the M value for acetylcholine receptor overlapping minimally with neurofilament (0.11±0.03; 6 NMJs, AChR on NF). A significant difference was detected using one-way ANOVA (P = 0.0002). Asterisks indicate significant difference against AChR on NF by Bonferroni post-test. Scale bars: A, 10 µm; B, C, 1 µm.

A commercial antibody against P/Q-type VDCCs stained NMJs of wild-type mice at postnatal day 15 in a punctate pattern (Fig. 1A). Importantly, these signals were absent in the NMJs of littermate P/Q-type VDCC knockout mice (*Cacna1a^−/−^)*, demonstrating the specificity of the immunohistochemistry signals (Fig. 1A). Three-dimensional reconstruction using confocal microscope z-stacks revealed that P/Q-type VDCCs distributed in a discrete punctate pattern within the NMJs labeled by α-bungarotoxin that binds specifically to acetylcholine receptors (Fig. 1B, C). In the orthogonal cross section view of the NMJ, P/Q-type VDCCs were detected at the bottom of the primary gutter of endplates, where motor nerve terminals reside (Fig. 1B).

Importantly, many punctate signals of P/Q-type VDCCs that overlapped with the α-bungarotoxin staining aligned with the punctate distribution pattern of the active zone protein Bassoon as shown in the top-down views and the orthogonal views of the confocal z-stacks (Fig. 1C_1–5_). The punctate staining pattern of P/Q-type VDCC and Bassoon were different from the diffuse immunohistochemical-staining pattern of the synaptic vesicle-associated protein SV2 [14]–[16], suggesting their localization at the active zones. This distribution pattern of P/Q-type VDCC and Bassoon in NMJs is consistent with our previous study demonstrating the binding of VDCCs and Bassoon [14]. Of the manually identified P/Q-type VDCC puncta that overlapped with α-bungarotoxin signal, 85% of the puncta had some overlapping Bassoon signal suggesting that Bassoon is binding to the cytosolic side of P/Q-type VDCC in presynaptic terminals (Fig. 1C_4_). Furthermore, colocalization of P/Q-type VDCC and Bassoon was indicated by the significantly higher Manders’ coefficient M values for Bassoon overlapping with P/Q-type VDCC (0.42±0.04) and P/Q-type VDCC overlapping with Bassoon (0.39±0.02) compared to the M value for acetylcholine receptor overlapping minimally with neurofilament (0.11±0.03) (Fig. 1C_6_). These data demonstrated that P/Q-type VDCCs localize at the presynaptic terminals of NMJs with the active zone protein Bassoon.

Interestingly, some P/Q-type VDCC signals were observed near but outside the acetylcholine receptor cluster (Fig. 1). This observation is compatible with the reported P/Q-type VDCC immunoreactivity in Schwann cells [10] and muscles [36].

### Bassoon Interaction Modulates Inactivation of VDCCs

P/Q-type VDCCs and Bassoon preferentially co-localized in the NMJs, and we previously demonstrated that these two proteins bind directly [14]. Thus, one potential role of this interaction at the presynaptic terminal of NMJs is to modify VDCC functions to enhance synaptic transmission. To test this hypothesis, we analyzed the calcium channel characteristics with or without Bassoon. A cell line stably expressing the P/Q-type VDCC (Cav2.1, ß1a, α2/δ) was transfected with a bicistronic vector expressing Bassoon and GFP. Transfected cells were identified by the GFP fluorescence and analyzed by whole-cell patch-clamp technique. Cells transfected with a bicistronic vector expressing only GFP were used as controls.

**Figure 2:**
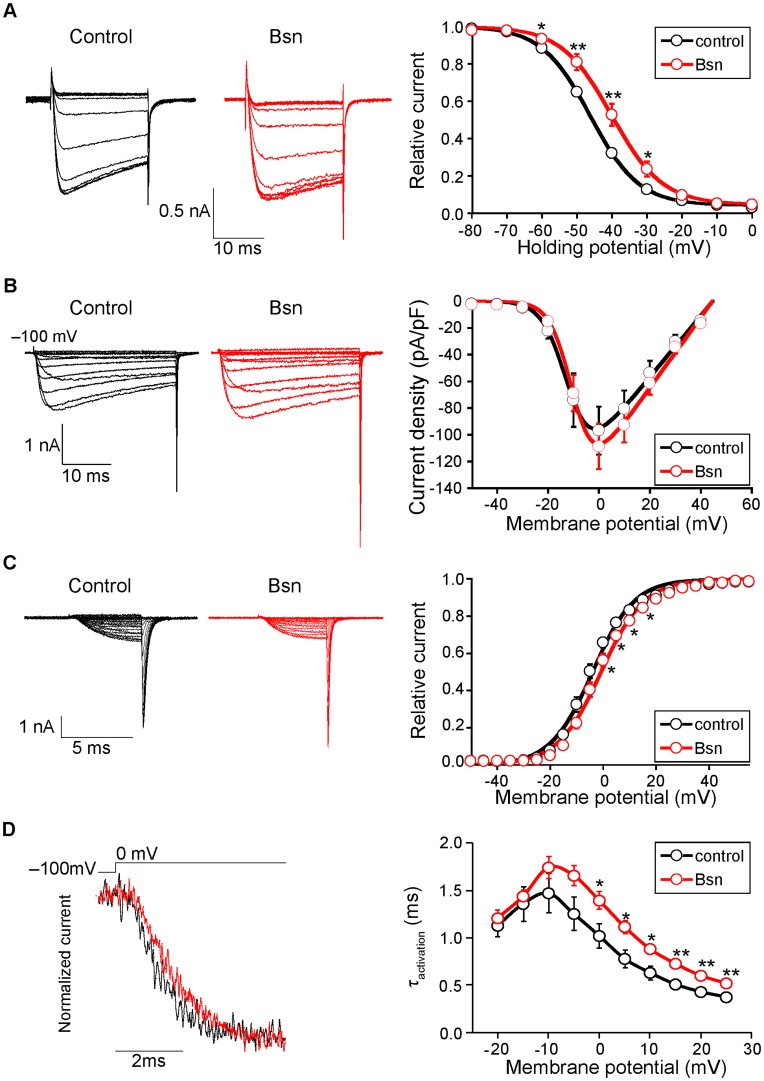
Inactivation properties of the P/Q-type VDCC were suppressed by Bassoon. (A) Left, inactivation of P/Q-type VDCC (Cav2.1) currents in BHK cells stably expressing VDCCs and transfected with an expression vector pBassoon-IRES2-GFP (Bsn, red) or an empty pIRES2-GFP vector (control, black). The peak amplitudes were normalized for Ba^2+^ currents elicited by 2-s pulses to 0 mV from a holding potential of –100 mV. Right graph shows inactivation curves for P/Q-type VDCCs with or without Bassoon. The half-inactivation potential was significantly higher in Bassoon expressing cells (–40.0±1.7 mV) compared to controls (–46.1±0.6 mV). (B) *I-V* relationships of P/Q-type VDCC showed no difference between with or without Bassoon. Left, representative traces are Ba^2+^ currents of Cav2.1 with or without Bassoon by applying test pluses from –100 mV (holding potential) to –50 mV up to 40 mV in 10 mV increments. Right graph shows current density-voltage (*I-V*) relationships. (C) The activation property of P/Q-type VDCCs in the presence of Bassoon exhibited a depolarization shift. Left, effects of Bassoon on activation of Cav2.1 currents elicited in BHK cells. Tail currents were elicited by repolarization to –60 mV after 5-ms test pulse from –50 to 55 mV with 5 mV increments. Right, activation curves were determined using these tail currents with or without Bassoon. The half-activation potential was significantly higher in Bassoon expressing cells (–0.7±1.1 mV) compared to controls (–4.0±1.1 mV). (D) Activation kinetics of P/Q-type VDCC currents. Left, tail currents were evoked by 5 ms depolarization from the holding potential (–100 mV) to 0 mV. Right graph shows the activation time constants (τ activation) with or without Bassoon. The activation time constant (τ) increased significantly in the presence of Bassoon at membrane potentials higher than 0 mV. Recordings from eight independent cells were averaged and the mean ± SEM are shown. Asterisks indicate significant difference compared to the controls, *p<0.05, **p<0.01.

The most prominent effect of Bassoon on VDCCs was observed on inactivation parameters. The inactivation property of P/Q-type VDCC was significantly suppressed by the co-expressed Bassoon (Fig. 2A, Table 1). The half-inactivation potential was shifted by 6.1 mV in Bassoon expressing cells (−40.0±1.7 mV) compared to controls (−46.1±0.6 mV). We observed a significant depolarizing shift in the voltage dependence of inactivation. This effect of Bassoon on P/Q-type VDCC is similar to the effect of Rim1 on VDCCs [17], [19].

**Table 1:** Effects of Bassoon on current density, activation, and inactivation of P/Q-type VDCC in BHK cells expressing Ca_v_2.1, α2/δ and ß1a^ 1) 2)^.

	Current density	Activation parameters			Inactivation parameters		
	(pA/pF) ^3)^	*V_0.5_* (mV)	*K* (mV)	τ (ms) ^4)^	*a*	*V_0.5_* (mV)	*K* (mV)
Control	−96.9±18.1	−4.0±1.1	7.5±0.3	1.02±0.13	0.96±0.01	−46.1±0.6	−7.0±0.2
	(22)	(12)	(12)	(12)	(8)	(8)	(8)
Bassoon	−108.8±16.9	−0.7±1.1	7.9±0.3	1.39±0.09	0.95±0.01	−40.0±1.7	−6.6±0.2
	(17)	(10)*	(10)	(10)*	(8)	(8)**	(8)

1)*P<0.05, **P<0.01 versus control.

2)Numbers of cells analyzed are indicated in the parenthesis.

3)Ba^2+^ currents evoked by depolarizing pulse from a *V*_h_ of −100 mV to 0 mV were divided by capacitance.

4)Activation time constants obtained from currents elicited by 5-ms test pulse to 0 mV. The activation phases are well fitted by a single exponential function.

The co-expression of Bassoon did not alter the current density-voltage (*I-V*) relationships of P/Q-type VDCCs (Fig. 2B, Table 1), but altered the activation properties and the voltage dependency of activation time constant. The half-activation potential was significantly higher in Bassoon expressing cells (−0.7±1.1 mV) compared to controls (−4.0±1.1 mV) (Fig. 2C). The activation time constants were slower in positive membrane potentials (Fig. 2D).

These results suggested that Bassoon might augment Ca^2+^ influx when P/Q-type VDCCs open by repetitive depolarization at NMJs.

### Exercise Suppresses Active Zone Loss in Aged NMJs

The electrophysiology data suggest the possibility that Bassoon plays a role in synaptic transmission at NMJs by enhancing the calcium influx through presynaptic P/Q-type VDCCs. In addition, Bassoon has been reported to play a role in synaptic vesicle trafficking to presynaptic membranes in central nervous system synapses [37]–[39]. These roles of Bassoon in VDCC modulation and synaptic vesicle trafficking suggest that NMJ synaptic transmission may be attenuated if presynaptic terminals lack Bassoon. In our recent study, we have discovered a condition that potentially causes such defect of NMJs. In aged mouse, Bassoon immunohistochemistry signal is significantly decreased in innervated, aged NMJs compared to NMJs of young adult mice [15]. Compatibly, aged NMJs exhibit stronger synaptic depression during repeated stimulation compared to young NMJs [40]. These findings raised the possibility that aged NMJ function may be attenuated because of the decreased level of Bassoon and prompted us to seek ways to ameliorate the Bassoon protein levels in aged NMJs. To this end, we utilized an exercise intervention because beneficial effects of exercise for the nervous system have been described previously [31]–[35].

In this study, we used our operant-based apparatus for isometric tongue force training in rats [41], [42]. We chose rats because, unlike mice, they are able to perform under the increased force requirements [41], [42]. The survival rate of Sprague-Dawley rats significantly reduces beyond two years of age [43]. Thus, rats were trained from 22-month-old for approximately two months under the increased tongue force conditions in the operant apparatus and were analyzed at 24-month-old to examine the aged NMJs. After the training period, genioglossus muscle of the tongue was analyzed because this muscle has been reported as one of the primary muscle for tongue protrusion [44]. First, the NMJ denervation rate was analyzed by labeling motor nerve terminals with antibodies for neurofilament and synaptic vesicle related protein SV2, and endplates with fluorescently labeled α-bungarotoxin. Most aged endplates exhibited matching motor nerve signals demonstrating innervation, which is consistent with the previous report [45]. Furthermore, the trained and untrained rats did not show a difference in innervation rate. On average, 94.0% of NMJs were innervated in trained-aged rats (3 rats), and 99.3% of NMJs were innervated in untrained-aged rats (2 rats).

**Figure 3:**
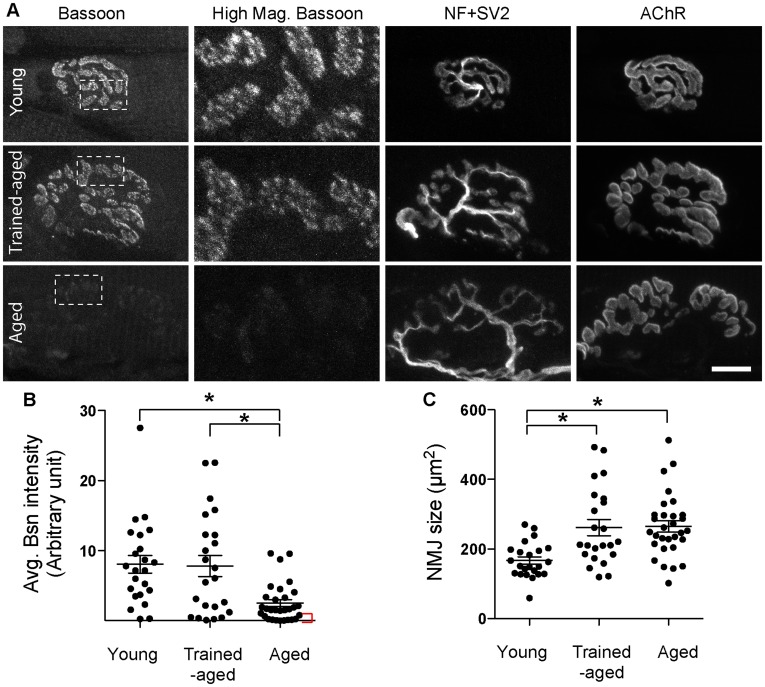
Exercise ameliorated the active zone protein Bassoon level in aged NMJs. (A) NMJs of trained aged rats (trained-aged) showed higher Bassoon signal intensity compared to the NMJs of un-trained aged rats (aged). Two-year-old rats underwent isometric strength training of tongue muscles for two months. Genioglossus muscles of the tongue were sectioned and stained with antibody against Bassoon (active zone marker), neurofilament and SV2 (nerve morphology, NF+SV2), and α-bungarotoxin (acetylcholine receptors, AChR). Highly magnified Bassoon staining of the area indicated by white dotted boxes are shown in the second column from the left (High Mag. Bassoon). NMJs of young rats (young, postnatal day 56) exhibited higher Bassoon signal intensity than aged rats, similar to our previous mouse study [15]. Scale bar: 10 µm. (B) Average signal intensity of Bassoon was significantly higher in NMJs of young rats and trained-aged rats compared to those of untrained-aged rats (mean ± standard error in arbitrary intensity units: young rats, 8.06±1.27; trained-aged rats, 7.81±1.51; and aged rats, 2.51±0.51; four rats in each group). The red bracket indicates a subgroup of NMJs in aged rats with a minuscule level of Bassoon signal. Young rats and trained-aged rats were not significantly different. (C) The NMJ size did not change significantly by the isometric strength training (trained-aged rats, 261.7±23.2 µm^2^; untrained aged rats, 264.8±16.5 µm^2^). Young rats had smaller NMJs (166.5±10.5 µm^2^) than the aged rats (trained and un-trained), which is consistent with the body and tissue size difference between these two ages. Quantifications in (B, C) are from four rats in each group (a total of 23 – 30 NMJs) and shown by scattered plot with the mean ± standard error by lines. Asterisks indicate significant differences by one-way ANOVA and Bonferroni’s multiple comparison post-test, P<0.05.

Next, the tongue muscles were analyzed for the active zone protein Bassoon level in NMJs by fluorescent immunohistochemistry and confocal microscopy, using our previously reported methods [15]. In NMJs of genioglossus muscles of young adult rats at postnatal day 56, the anti-Bassoon antibody stained a punctate staining pattern similar to mouse NMJs (Fig. 3). In the NMJs of aged-rats (two-year-old) genioglossus muscles, the average Bassoon signal intensity was significantly lower than that of young adults (Fig. 3A, B). This result demonstrated that similar decline of the active zone protein occur in NMJs of two mammalian species, rats and mice.

In contrast to these NMJs of untrained-aged rats, NMJs of trained-aged rats had significantly higher mean signal intensity of Bassoon staining (mean ± standard error in arbitrary intensity units: trained-aged rats, 7.81±1.51; untrained-aged rats, 2.51±0.51; four rats in each group) (Fig. 3B). The important difference between trained and untrained group is the abundance of NMJs with minuscule level of Bassoon signals in untrained aged rats (indicated by the red bracket in Fig. 3B), which was attenuated in the trained-aged rats. The mean signal intensity of Bassoon in trained-aged rats was similar to that of the young adults. However, the training had no effect on the NMJ size (Fig. 3C; trained-aged rats, 261.7±23.2 µm^2^; untrained-aged rats, 264.8±16.5 µm^2^; no significant difference). Thus, the difference of Bassoon signal intensity is not due to the innervation rate or the NMJ size. To assess the functional effect of training, we compared the peak tongue force of trained-aged rats after the entire training session versus the untrained-aged rats under a minimal force condition. The peak force value was greater in the trained-aged rats (17.5±1.8 grams) compared to the untrained-aged rats (14.7±0.7 grams), but they did not differ significantly. These results demonstrated that isometric strength training ameliorates the loss of active zone protein Bassoon at aged NMJs, which may aid the functional preservation of synaptic transmission.

## Discussion

The subsynaptic location of P/Q-type VDCCs within a NMJ and their functional modification by the interaction with the active zone protein Bassoon were not known previously. In this study, we show that P/Q-type VDCCs localized in a discrete punctate pattern in the presynaptic terminals of NMJs and co-localized with the Bassoon. We have previously shown that P/Q-type VDCCs and Bassoon interact [14], and in this study we demonstrated that this interaction suppresses the inactivation of VDCCs. The ability to modify VDCC function by Bassoon suggests that a loss of active zone proteins has the potential to impair the synaptic transmission of NMJs. This impairment may occur in aged NMJs because we have recently shown that aged mouse NMJs exhibit decreased level of Bassoon [15] and we show in this study that aged rats exhibit the same decrease. In an attempt to ameliorate the loss of active zone proteins in aged NMJs, we subjected the aged rats to isometric tongue force training sessions. Importantly, this strength training was able to significantly ameliorate the loss of Bassoon in aged rat NMJs.

### P/Q-type VDCCs Localize at the NMJ Active Zones

P/Q-type VDCCs have been detected at rodent NMJs by immunohistochemistry [9]–[12] and by electrophysiology [1], [2], [46]. These immunohistochemistry analyses of moderately magnified en face views or a transverse section of NMJs showed diffusely distributed P/Q-type VDCCs throughout the presynaptic membrane. However, these studies have not compared the location of P/Q-type VDCCs against the active zones. Here, we revealed for the first time that P/Q-type VDCCs localize preferentially with the active zone protein Bassoon in NMJs, which suggests that these co-localization spots are the active zones. The P/Q-type VDCC distributed in a discrete punctate pattern like the active zone proteins. The reason for the difference in detected patterns between the current data and the published data is unknown, but the results in this study are in good agreement with the assumed accumulation of P/Q-type VDCCs in close vicinity of active zones based on electrophysiological analyses [2].

Furthermore, accumulation of P/Q-type VDCCs at the active zones is consistent with the molecular mechanism of active zone organization that we proposed in our previous studies. We demonstrated that muscle-derived synapse organizer laminin ß2 binds to the extracellular side of P/Q-type VDCC and organizes the active zones [16]. The P/Q-type VDCC functions as a scaffolding protein to link this extracellular interaction to cytosolic active zone proteins by the interactions between VDCC ß-subunits and active zone proteins [14], [47]. Thus, alignment of P/Q-type VDCCs and Bassoon in three dimensions is compelling data supporting this molecular mechanism to organize NMJ active zones. Similarly, in Drosophila NMJs, VDCC (Cacophony) distributes in a punctate pattern and aligns with the active zone protein Bruchpilot [21], [48].

### Bassoon Interaction Modulates Inactivation of VDCCs

The inactivation property of P/Q-type VDCCs was significantly suppressed by Bassoon co-expressed in the same cell. Previously, we have demonstrated that Bassoon binds to P/Q-type VDCC through the VDCC ß subunit [14]. Thus, the effect of Bassoon on P/Q-type VDCC is likely to occur through the binding of Bassoon and the VDCC complex. If Bassoon is associated with P/Q-type VDCCs opened by repetitive depolarization, Bassoon suppresses the inactivation of the VDCCs and causes the channels to open longer. Therefore, Ca^2+^ influx through P/Q-type VDCCs will be augmented by Bassoon. Such modification of the VDCC function by Bassoon suggests a possibility that Bassoon may augment Ca^2+^ influx when presynaptic P/Q-type VDCCs open by repetitive action potentials and may contribute to NMJ synaptic transmission.

Our current results are in agreement with the study of Frank and colleagues who showed that Bassoon increases the Ca^2+^ influx through L-type VDCCs into inner hair cells of the auditory system [39]. Furthermore, the functional modification of P/Q-type VDCC by Bassoon is similar to modification by another active zone protein Rim1, which binds to the VDCC ß subunits and suppresses the inactivation property of P/Q-type VDCC [17], [19]. Thus, Bassoon has the potential to modulate synaptic transmission efficiency by binding to presynaptic VDCC complexes and modifying the channel function, in addition to the reported role in accumulating synaptic vesicles to the presynaptic membrane [37]–[39].

Does the functional modification of VDCC contribute to active zone formation? The synapse organizer laminin ß2 induces presynaptic differentiation in cultured motor neurons despite P/Q- and N-type VDCC blockade by specific toxins, providing evidence for dispensability of Ca^2+^ influx into nerve terminals for active zone formation [16]. This finding suggests that ion channel activity or the modification of channel function may not be required for active zone formation.

### Suppression of Active Zone Loss in Aged NMJs by Exercise

We have detected active zone impairment in aged NMJs of two mammalian species: rats and mice. A decreased number of active zones has been shown to impair NMJ synaptic functions in humans and mice [2], [49], [50]. At these aged NMJs, a reduced Bassoon protein level has the potential to decrease Ca^2+^ influx into the presynaptic terminals and to weaken synaptic transmission (based on our electrophysiological analyses explained in the previous section), in conjunction with an impairment of synaptic vesicle trafficking to presynaptic membranes [37]–[39]. These views are compatible with the attenuation of synaptic function in aged NMJs compared to young adult NMJs, such as, the stronger synaptic depression during repeated stimulation [40], the reduced end plate potential amplitude (plateau level) after repetitive stimulation [25], and the reduced frequency of miniature end-plate potential [25], [26]. Thus, active zone loss seems to be a part of NMJ impairment in aging.

Here, we tested whether this impairment can be ameliorated by exercise. Beneficial effects of exercise intervention for the nervous system have been described previously, such as neurotrophin upregulation [31]–[33], [35] and recovery from nerve injury [34]. However, this is the first demonstration of a beneficial effect on the presynaptic active zones by exercise intervention. In this study, we chose isometric strength training because, if applied to humans, this type of training is practical for the elderly compared to the aerobic type training. Propitiously, the training ameliorated the loss of Bassoon at NMJs. Interestingly, the trained rats produced stronger tongue force after the training compared to the age matched controls. However, this difference could be attributed to the force set during the training sessions and requires further investigation to reveal the functional difference between the trained and control groups. Importantly, the improvement of active zone proteins at aged rat NMJs after training is in agreement with the study by Fahim who showed electrophysiologically that NMJ function improves after endurance training of aged mice [27]. In summary, preserving the active zone organization at aged NMJs is likely to have a positive impact on the function of NMJs.

## Materials and Methods

### Ethics Statement

All animal studies have been approved by the University of Kansas Medical Center Institutional Animal Care and Use Committee. This study was carried out in strict accordance with the recommendations in the Guide for the Care and Use of Laboratory Animals of the National Institutes of Health.

### Animals

C57BL/6 mice (Jackson Laboratory), P/Q-type VDCC knockout mice on C57BL/6 background (*Cacna1a^−/−^*), and Sprague-Dawley rats (male retired breeders, Harland) were maintained at the University of Kansas Medical Center animal facility. Rodents were maintained on a 12/12 hr. light/dark cycle and the behavior training was performed during the light portion of this cycle. The generation of *Cacna1a^−/−^* mice and the absence of mRNAs and proteins for the *Cacna1a* gene have been described previously [51].

### Antibodies

Following antibodies were used: Bassoon (SAP7F407; Enzo Life Sciences), neurofilament (SMI312, Covance), P/Q-type VDCC (152103, Synaptic systems), SV2 (Developmental Studies Hybridoma Bank), Alexa Fluor 488-, 568-, 647-conjugated secondary antibodies and α-bungarotoxin (Invitrogen).

### Isometric Tongue Strength Training

The rats were placed on a gradual water restriction schedule and underwent isometric strength training for approximately two months starting at 22-months of age using an operant apparatus that has been described in detail previously [42]. This apparatus has been used successfully in our recently study of orolingual motor deficits in the rat model of ALS [41]. Rats licked water from the top of an isometric force-sensing disc at a distance of two mm above the disc. Water was delivered to the disc when four licks were made that met or exceeded a programmed force criterion. The rats readily engaged in the task, emitting licks throughout the 6-minute training sessions. After fourteen days of baseline training under a one-gram force requirement, the force criterion was increased to 15 grams for seven days, 20 grams for seven days, and then to 30 grams for ten days. Rats were trained for one session/day, four days a week, until the final 30-gram phase. Rats were tested twice/day, four days a week during the ten days of 30-gram force requirement. The untrained control group was comprised of age-matched rats that received similar amounts of water and were housed in the same environment for the same duration as the trained group. At the completion of training, peak forces of trained and untrained rats were measured under a low-force (5 grams) condition.

### Expression Plasmid

A cDNA encoding the C-terminal domain of mouse Bassoon (*Bsn*) (1053 amino acids) was PCR amplified from a cDNA clone mKIAA0434 (353–3514 base pairs; Kazusa DNA Research Institute) and subcloned into a bicistronic expression vector pIRES2-GFP (Clontech, pBassoon-IRES2-GFP). We have previously shown the direct binding of a recombinant protein of the same domain of Bassoon with P/Q-type VDCC ß subunit using co-immunoprecipitation analysis [14].

### Cell Line and Transfection

Baby hamster kidney (BHK) cells (American Type Culture Collection) stably expressing Cav2.1, α2/δ and ß1a (BHK6-2) were described previously [17]. This BHK cell line was transfected with an expression vector pBassoon-IRES2-GFP or an empty pIRES2-GFP vector, using SuperFect Transfection Reagent (QIAGEN). The electrophysiological measurements were performed 48–72 h after transfection.

### Electrophysiology

Whole-cell patch-clamp technique was performed on BHK cells at 22–25°C, as previously described [17]. Electrophysiological measurements were performed on BHK cells at room temperature (22–25 °C) using whole cell mode of the patch-clamp technique with an EPC-10 (List Medical Electronics, Darmstadt, Germany). Patch pipettes were made from borosilicate glass capillaries (1.5 mm, outer diameter; Hilgenberg, Malsfeld, Germany) using a model P-97 Flaming-Brown micropipette puller (Sutter Instrument Co., San Rafael, CA). The patch electrodes were fire-polished. Pipette resistance ranged from 1 to 2 megohm when filled with the pipette solutions described below. The series resistance was electronically compensated to >70% and both the leakage and the remaining capacitance were subtracted by −P/4 method. Currents were sampled at 100 kHz after low pass filtering at 10 kHz (−3 db) using the 8-pole Bessel filter (Model 900, Frequency Devices, Haverhill, MA) in the experiments of activation kinetics, otherwise sampled at 10 kHz after low pass filtering at 2 kHz (−3 db). Data were collected and analyzed using the Pulse 10 software (Heka, Lambrecht, Germany). Ba^2+^currents were recorded in an external solution that contained (in mM): 3 BaCl_2_, 155 tetraethylammonium chloride, 10 HEPES, 10 glucose (pH adjusted to 7.4 with tetraethylammonium-OH). The pipette solution contained (in mM): 95 Cs-aspartate, 40 CsCl, 4 MgCl_2_, 5 EGTA, 2 ATPMg, 5 HEPES, 10 creatine phosphate (pH adjusted to 7.4 with CsOH).

### Voltage Dependence of Inactivation

To determine the voltage dependence of inactivation (inactivation curve) of VDCC, Ba^2+^ currents were evoked by 20-ms test pulse to 10 mV after the 10-ms repolarization to –100 mV following 2-s *V*_h_ displacement (conditioning pulse) from –100 mV to 40 mV with 10 mV increments. Amplitudes of currents elicited by the test pulses were normalized to those elicited by the test pulse after a 2-s *V*_h_ displacement to –100 mV. The mean values were plotted against potentials of the 2-s *V*_h_ displacement. The inactivation curve was monophasic, the mean values were fitted to the single Boltzmann’s equation: *h*(*V*_h_)  =  (1–*a*)+*a/*{1+exp[(*V*_0.5_–*V*_h_)/*k*]}, where *a* is the rate of inactivating component, *V*_0.5_ is the potential to give a half-value of inactivation, and *k* is the slope factor.

### Voltage Dependence of Activation

Tail currents were elicited by repolarization to –60 mV after 5-ms test pulse from –50 to 55 mV with 5 mV increments. Amplitude of tail currents were normalized to the tail current amplitude obtained with a test pulse to 55 mV. The mean values were plotted against test pulse potentials, and fitted to the Boltzmann’s equation: *n*(*V*_m_) = 1/{1+exp[(*V*_0.5_–*V*_m_)/*k*]}, where *V*_m_ is membrane potential, *V*_0.5_ is the potential to give a half-value of conductance, and *k* is the slope factor.

### Immunohistochemistry and Confocal Microscopy

The immunohistochemistry and confocal microscopy methods have been described previously [14], [15]. In brief, mice and rats were fixed by transcardiac perfusion with 4% paraformaldehyde in PBS. Muscles were post-fixed in the same fixative at room temperature for 20 minutes, cryoprotected in 20% sucrose/PBS, frozen in Optimal Cutting Temperature compound (Sakura). Longitudinal sections were cut using a cryostat at a thickness of 20 µm and blocked in PBS containing 2% BSA, 2% normal goat serum, and 0.1% Triton. Sections were sequentially incubated with primary antibodies overnight, washed with PBS, and incubated in a mixture of Alexa Fluor (488 or 568)-conjugated secondary antibodies and Alexa Fluor (594 or 647)-conjugated α-bungarotoxin for 2 hours at room temperature. After washing, the sections were mounted with Vectashield (Vector). No staining was observed when primary antibodies or Alexa Fluor-conjugated α-bungarotoxin were omitted. Sequentially scanned confocal Z-stacks were obtained using a Nikon C1Si confocal microscope (Nikon, Tokyo, Japan) with a 100× objective lens (Apo TIRF, NA = 1.49).

### Denervation Analysis

The denervation analysis methods have been described previously [41]. In brief, the muscle cryostat sections were stained with antibodies against nerves (neurofilament and SV2) and endplates (Alexa594-α-bungarotoxin to label acetylcholine receptors). Adult motor nerve terminals show perfect overlap with endplates, which shows innervated NMJs. Any non-occupied area of the endplates, whether in part (partial innervation) or in full (full denervation), were judged as denervation. Quantifications are from three trained-aged rats and two un-trained rats (a total of 24 – 31 NMJs per animal).

### Image Analysis

Three-dimensional reconstruction of confocal Z-stacks and image analyses were conducted using Metamorph (Universal Imaging Corporation).

Colocalization analyses were performed on maximal projected confocal images. First, the degree of overlap between Bassoon signals and P/Q-type VDCC signals was analyzed by manually identifying Bassoon and VDCC puncta that overlap with α-bungarotoxin signal and by manually evaluating the overlap between the two signals. Next, the degree of overlap between Bassoon and P/Q-type VDCC signals was analyzed by Manders’ coefficients with automatic thresholds using ImageJ (W. Rasband, National Institutes of Health, http://rsb.info.nih.gov/ij/) and JACoP plugin [52]. This analysis method was chosen because the signal intensities of these two antigens were different. A Manders’ coefficient provides a value (M) ranging from 0 to 1, with 1 standing for complete colocalization and 0 for no overlap between the two images. We tested this analysis method using images of NMJs stained with an anti-neurofilament antibody and α-bungarotoxin that exhibit minimal overlap. The average M value was −0.13±0.04 (n = 6 NMJs), which showed the absence of correlation and the validity of this analysis method.

For the quantification of the NMJ size, areas of α-bungarotoxin-labeled acetylcholine receptor (AChR) clusters were measured from maximal-projected confocal images using the autothreshold function, “threshold for light objects”. For the quantification of the Bassoon immunohistochemistry signal (without level adjustment) in the NMJs, we restricted the measurements to presynaptic terminals overlying α-bungarotoxin-labeled AChR clusters and quantified only the fully innervated NMJs that were stained by anti-neurofilament plus anti-SV2 antibodies. The Bassoon signal intensity in NMJs of young rats, trained-aged rats, and untrained-aged rats were quantified using a unified threshold value, which was manually determined by fitting the threshold to the punctate staining pattern of Bassoon in NMJs. The average Bassoon signal intensity was determined by dividing the total Bassoon signal intensity in a single NMJ by the NMJ size. Quantifications are from four rats in each group (a total of 23 – 30 NMJs per group). Muscle fibers exhibit slow or fast fiber type characteristics, and NMJs differ in morphology and size between these two fiber types [53]–[55]. However, in this study, we did not differentiate the NMJs on the basis of fast or slow fibers because the density of the active zone does not change between these fiber types [13].

### Statistics

Statistical significance was assessed by an unpaired t-test for electrophysiological analysis, and by 1-way ANOVA with Bonferroni’s multiple comparison post-test for the immunohistochemistry signal quantification, using GraphPad Prism software.
